# The association between RGS4 and choline in cardiac fibrosis

**DOI:** 10.1186/s12964-020-00682-y

**Published:** 2021-04-23

**Authors:** Jing Guo, Pengzhou Hang, Jie Yu, Wen Li, Xiuye Zhao, Yue Sun, Ziyi Fan, Zhimin Du

**Affiliations:** 1 Institute of Clinical Pharmacology, The Second Affiliated Hospital of Harbin Medical University (The University Key Laboratory of Drug Research, Heilongjiang Province), Harbin, 150086 People’s Republic of China; 2 Department of Clinical Pharmacology, College of Pharmacy, Harbin Medical University, Harbin, 150081 People’s Republic of China; 3 State Key Laboratory of Quality Reserch in Chinese Medicines, Macau University of Science and Technology, Macau, Macau, 150086 People’s Republic of China

**Keywords:** RGS4, Cardiac fibrosis, Choline, TGF-β1

## Abstract

**Background:**

Myocardial fibrosis is caused by the adverse and powerful remodeling of the heart secondary to the death of cardiomyocytes after myocardial infarction. Regulators of G protein Signaling (RGS) 4 is involved in cardiac diseases through regulating G protein-coupled receptors (GPCRs).

**Methods:**

Cardiac fibrosis models were established through cardiac fibroblasts (CFs) treatment with transforming growth factor (TGF)-β1 in vitro and mice subjected to myocardial infarction in vivo. The mRNA expression of RGS4, collagen I/III and α-SMA detected by qRT-PCR. Protein level of RGS4, collagen I, CTGF and α-SMA detected by Western blot. The ejection fraction (EF%) and fractional shortening (FS%) of mice were measured by echocardiography. Collagen deposition of mice was tested by Masson staining.

**Results:**

The expression of RGS4 increased in CFs treatment with TGF-β1 and in MI mice. The model of cardiac fibrosis detected by qRT-PCR and Western blot. It was demonstrated that inhibition of RGS4 expression improved cardiac fibrosis by transfection with small interfering RNA in CFs and injection with lentivirus shRNA in mice. The protective effect of choline against cardiac fibrosis was counteracted by overexpression of RGS4 in vitro and in vivo. Moreover, choline inhibited the protein level of TGF-β1, p-Smad2/3, p-p38 and p-ERK1/2 in CFs treated with TGF-β1, which were restored by RGS4 overexpression.

**Conclusion:**

This study demonstrated that RGS4 promoted cardiac fibrosis and attenuated the anti-cardiac fibrosis of choline. RGS4 may weaken anti-cardiac fibrosis of choline through TGF-β1/Smad and MAPK signaling pathways.

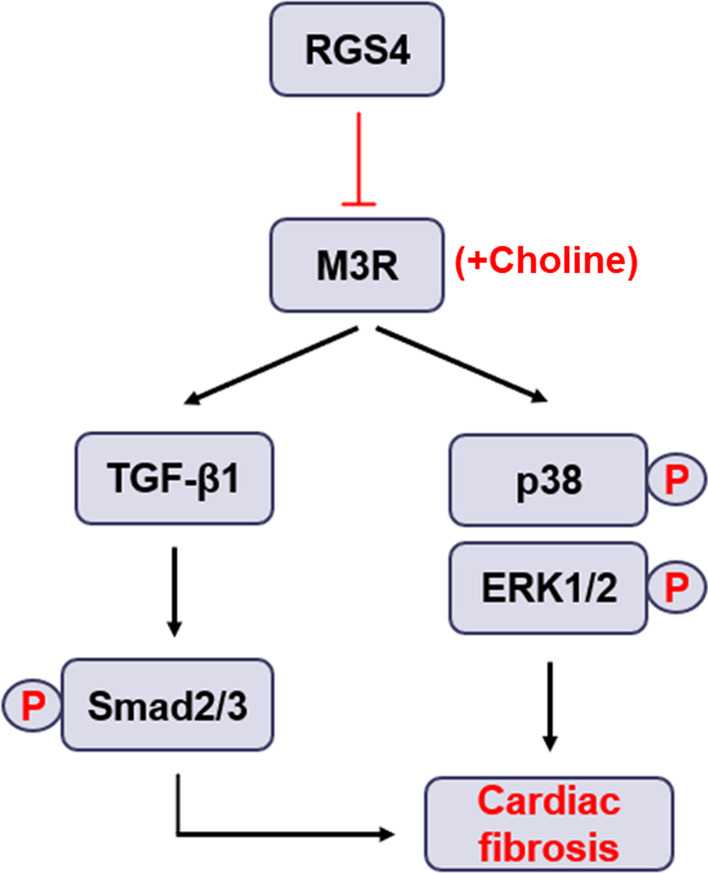

**Video Abstract: Video Byte of this article**

**Supplementary Information:**

The online version contains supplementary material availlable at 10.1186/s12964-020-00682-y.

## Background

Ischemic heart disease (IHD) is a worldwide problem threatening human health due to its high morbidity and mortality. One of the characteristics of IHD is cardiac fibrosis which is caused by the adverse remodeling of the heart secondary to the death of cardiomyocytes after myocardial infarction (MI) [1, 2]. Cardiac fibroblasts activate in response to the loss of cardiomyocytes that create a lesion in myocardium and proliferate to fill and repair the lesion. In order to maintain the structural integrity of the heart, extracellular matrix (ECM) components, especially collagen types I and III, are over secreted and deposited in myocardium [3, 4]. Yet, such a protective mechanism is in the face of creating a state of ECM overproduction when ECM homeostasis is dysregulated, leading to disordered cardiac fibrosis and the associated deterioration of heart function toward ultimate heart failure [5–7].

Regulators of G protein signaling (RGS proteins) are closely related to signal transduction via G protein-coupled receptors (GPCRs) that are the key signaling mediators of many cellular functions and pathological processes [8]. RGS proteins are identified as negative regulators of G protein signal system [9, 10]; they act as GTPase-activating proteins (GAPs) to limit G protein activity via accelerating the rate of Gα-GTP hydrolysis [8, 11]. Previous studies revealed that several members of RGS protein family are involved in multiple heart diseases. For example, RGS14 could regulate cardiac remodeling through the MEK-ERK1/2 signaling pathway [12]. Cardiac-specific overexpression of RGS5 in transgenic mice effectively alleviates the damage caused by cardiac hypertrophy and fibrosis [11]. RGS6 is involved in cardiac hypertrophy through apoptosis signal-regulating kinase 1 [13]. RGS4 regulates atrial fibrillation caused by excessive exercise via modulating vagal sensitivity [14]. It suggests that RGS4 may be involved in the occurrence and development of cardiac diseases, so this study will explore the regulatory process of RGS4 in cardiac fibrosis.

Choline is a precursor of acetylcholine, which can activate muscarinic and nicotinic receptors, both of which belong to GPCRs [15]. Choline has been shown to have a variety of cardioprotective effects. For example, it confers cardiomyocytes the ability to tolerate cardiac ischemic injury [16]. Choline also protected cardiomyocytes from ischemia injury and oxidative stress [17]. A number of studies from our group and others have confirmed that choline plays a protective role in many heart conditions, such as myocardial ischemia/reperfusion injury, cardiac fibrosis and cardiac hypertrophy [18–20]. Choline, as an agonist of G protein coupled receptor, may interact with RGS protein in myocardial fibrosis. However, whether RGS4 antagonizes the effects of choline in cardiac fibrosis remain unclear.

## Materials and methods

### Neonatal mouse cardiac fibroblasts isolation and culture

All animal care and laboratory procedures of this study were approved by the Ethical Committee of Harbin Medical University and all animal were obtained from the Experimental Animal Center of Second Affiliated Hospital of Harbin Medical University, China. Neonatal mouse (1–3 days old) hearts were prepared for finely minced and placed together in 0.25% trypsin to get single cell suspension. After the cells were filtered and centrifuged (1000 rpm/min, 5 min) and then resuspended in DMEM (HyClone, USA) containing 10% fetal bovine serum and 5% penicillin/streptomycin. Finally, the cells were plated into Petri dishes (60 mm) or other specifications of culture plate and cultured under a condition of 5% CO_2_ and 95% air at 37 °C for 1.5 h. Then, cardiac fibroblasts preferential attached the bottom of petri dishes and new DMEM containing 10% fetal bovine serum and 5% penicillin/streptomycin was replaced in the Petri dishes to culture primary cardiac fibroblasts. The cardiac fibroblasts were pre-stimulated with choline (5 mM) for 1 h and then incubated with recombinant human TGF-β1 (20 ng/mL, Sigma-Aldrich Co., LLC, USA) for 48 h. Choline (1 mM) treatment was operate as describe previously [19]. The cells were used for the experiments.

### Mouse models of MI

Healthy male Kunming mice weighing about 20–25 g were kept under standard animal housing conditions which temperature is 23 ± 1 °C and humidity is 55 ± 5% with a 12/12 h light/dark cycle. Mice were anaesthetized with 2, 2, 2-Tribromoethanol (Sigma, USA) via i.p. (20 mg/kg), the skin of the chest was shaved and disinfected. Then the mice were intubated and ventilated with an artificial respiration machine (mouse ventilator, PhysioSuite, Kent Scientific corporation, USA) at a respiratory rate of 120 breaths/min and a tidal volume of 1.50 mL. An incision was performed between the 3th and the 4th rib space and the heart was exposed. Then, the left anterior descending coronary (LAD) artery was ligated with 8/0 silk thread. The chest cavity was sutured by 3/0 sutures and the thorax was closed. Sham-operated mice underwent the same surgical procedure but LAD artery without ligation. Choline treatment (14 mg/kg) was performed three days after MI, as described previously [19]. Four weeks later, the mice were killed after anesthesia and heart tissue was collected for subsequent experiments.

### In vivo lentivirus infection

The lentivirus-mediated shRNA for Rgs4 (LV-shRGS4) (5′ CACC GGGA GGTT CACA TCCT AAAC GAAT TTAG GATG TGAA CCTCCC3′) and the lentivirus carrying scrambled shRNA (LV-shNC) (5′GTTC TCCG AACG TGTC ACGT3′) as negative control were synthesized by Genechem (Shanghai, China). The lentivirus-mediated Rgs4 (LV-RGS4) was used to overexpress Rgs4. Briefly, mice were anaesthetized, a thoracotomy was performed through the fourth intercostal space. The ascending aortic artery and the main pulmonary artery were clamped. The lentivirus was injected (2.5 × 10^7^ TU·mL^−1^ at a volume of 100 μL) through the tip of the heart into the left ventricular cavity. The arteries were occluded for 10 s after lentivirus injection [21]. Myocardial infarction operation was started 7 days after lentivirus injection. The treatment process of mice was shown in Table 1.

**Table 1:** The treatment process of mice

	0 day	7 day	10 day	35 day
Part I	Lentivirus injection (LV-shRGS4/NC)	MI surgery	–	Collected heart tissue
Part II	Lentivirus injection (LV-RGS4/NC)	MI surgery	Choline treatment	Collected heart tissue

### Echocardiography

Four weeks after MI operation, cardiac function of mice was detected by echocardiography. Left ventricular ejection fraction (EF) and left ventricular shortening score (FS) were measured. The process was as described previously [19].

### Masson’s trichrome staining

Hearts were quickly dissected and immersed in 4% neutral buffered formalin for 24 h and stained with Masson’s trichrome according to the manufacturer's instructions. The extent of collagen deposition was calculated with image analysis software and the results are shown as the percentage of area occupied by fibrosis to the total area.

### Hematoxylin/eosin (H&E) staining

Hearts were quickly dissected and immersed in 4% neutral buffered formalin for 24 h and stained with Hematoxylin/Eosin according to the manufacturer's instructions.

### Transfection procedures

Prior to cell transfection, the medium of primary cardiac fibroblasts was replaced by serum-free DMEM to starve for 4–6 h. And then transiently transfected with RGS4 siRNA (50 nM), siRNA negative control (siNC) (50 nM), RGS4 plasmid (1 µg/mL) or empty plasmid (pcDNA3.1, 1 µg/mL) using Opti-MEMI (Invitrogen, USA) and X-treme GENE siRNA transfection reagent (Roche, Penzberg Germany) according to the manufacturer’s protocols. After 6 h, fresh medium containing 10% FBS was added to the Petri dishes and the cells were maintained in the culture medium for 48 h until the subsequent experiments. The RGS4 siRNA sequence was 5′-CCUCAAGUCUCGAYYCUACTT-3′. The siNC sequence was 5′-UUCUCCGAACGUGUCACGUTT-3′. SiRNA, siNC, RGS4 plasmid and empty plasmid (pcDNA3.1) were synthesized by GenePharma Co., Ltd (Shanghai, China).

### Cell viability assay

The cardiac fibroblasts were seeded in 96-well culture plates. The MTT assay (Amresco, Solon, USA) was performed when the number of adherent cells reached 2 × 10^4^ cells per well. 3-(4,5-dimethylthiazol-2-yl)-2,5-diphenyltetrazolium bromide (MTT) was used to test cell viability according to the manufacturer's protocols. The absorbance was calculated at 490 nm by Microplate Reader (Infinite M200, TECAN).

### Immunofluorescence staining

The cardiac fibroblasts were seeded on sterile glass cover slips in 24-well culture plates. Briefly, treated cardiac fibroblasts were washed with cold PBS buffer three times and then fixed with 4% paraformaldehyde for 15 min. The cell membrane was permeabilized by 0.4% Triton X-100 for 1 h and blocked with normal goat serum diluent for 1 h at 37 °C. The normal goat serum diluted in PBS at a ratio of 1:1. The cells were incubated with anti-α-SMA antibody (Abcam Inc., USA, 1:100) and anti-vimentin antibody (Bioss, China, 1:100) overnight at 4 ℃ and followed by incubation with a FITC-conjugated goat anti-rat antibody (ZSGB-Bio, China, 1:500) for 2 h at 37 ℃. After three times washed with PBS buffer, DAPI (Sigma-Aldrich, 1:50) was added into the well incubating for 5 min to stain the nuclei and then washed with PBS buffer for three times. Laser scanning confocal microscope (Olympus, Fluoview1000, Tokyo, Japan) was used to view the fluorescence staining.

### Western blot

The cultured cells or cardiac tissue were washed with PBS buffer and lysed in RIPA buffer (Beyotime, Jiangsu, China) supplemented with protease inhibitor and phosphatase inhibitors mixture. The concentration of total proteins was determined with BCA Protein Assay Kit (Beyotime, Shanghai, China). Protein sample (70 μg) was fractionated by SDS-PAGE (10% polyacrylamide gels) and transferred to nitrocellulose membrane. The nitrocellulose membranes were blocked in 5% nonfat milk PBS at room temperature for 2 h and then incubated overnight at 4 °C with primary antibodies for collagen I (1:500), CTGF (1:500), α-SMA (1:1000), RGS4 (1:200), M3R (1:500), TGF-β1 (1:500), total Smad2/3 (t-Smad2/3;1:1000), phosphorylated Smad2/3 (p-Smad2/3; 1:1000), t-ERK (1:1000), p-ERK (1:1000), t-p38 (1:1000), p-p38 (1:1000), β-actin (1:1000, ZSGB-Bio, China) and GAPDH (1:1000, ZSGB-Bio, China) followed by incubation with IRDye secondary antibodies (LI-COR, USA) at room temperature for 1 h. Antibodies against α-SMA, t-Smad2/3, p-Smad2/3, TGF-β1, t-p38, p-p38, t-ERK1/2 and p-ERK1/2 were purchased from Cell Signaling Technology (CST, USA). Antibodies against CTGF was purchased from Proteintech (China). Antibodies against RGS4 was purchased from Santa (USA). The immunoreactivity was detected using Odyssey CLx Infrared Imaging System (LI-COR Biosciences, Lincoln, NE, USA). The bands of each group were quantified by measuring the band intensity with Odyssey CLx version 2.1. The data was normalized to GAPDH and β-actin as an internal control. We showed full protein band in the Additonal file 4.

### RNA extraction and real-time PCR

Total RNA was extracted from cultured cardiac fibroblasts or heart tissue using Trizol reagent (Invitrogen, USA) according to manufacturer’s instructions. The RNA was then reverse-transcribed into complementary DNA. The mRNA expression was detected using SYBR Green incorporation on Roche Light-Cycler 480 Real Time PCR system (Roche, Germany), while GAPDH was used as an internal control. The sequences of primers used were listed as follows: RGS4: forward (F): 5′-GCCATGCAGGCTAAGAAAGGA-3′ and reverse (R): 5′-CCCTGGCTATTCTCCGCCAA-3′; collagen I: forward (F): 5′-ATCAGCCCAAACCCCAAGGAGA-3′ and reverse (R): 5′-CGCAGGAAGGTCAGCTGGATAG-3′; collagen III: forward (F): 5′-TGATGGGATCCAATGAGGGAGA-3′ and reserve (R): 5′- GAGTCTCATGGCCTTGCGTGTTT-3′; α-SMA: forward (F): 5′-GTCCCAGACATCAGGGAGTAA-3′ and reserve (R): 5′-TCGGATACTTCAGCGTCAGGA-3′; GAPDH forward (F): 5′-GGAAAGCTGTGGCGTGAT-3′ and reserve (R): 5′-AAGGTGGAAGAATGGGAGTT-3′. Quantitative real-time PCR was performed in 20 μL volumes with SYBR Green PCR Master Mix (Roche, USA) at 95 °C for 10 min and 40 cycles at 95 °C for 15 s, 60 °C for 30 s and 72 °C for 30 s, using Light Cycler 480 (Roche, USA). The amount of target (2^−ΔΔCT^) was calculated by normalizing to endogenous reference and relative to an average of the control samples.

### Determination of oxidative stress

The contents of malondialdehyde (MDA) and the activity of super oxide dismutase (SOD) were examined with Biochemical Analysis Kits (Jiancheng Biotechnology Co., Nanjing, China) in the light of the respective manufacturers’ protocols.

### Statistical analysis

Data are presented as the mean ± SEM. Student’s t-test was used for two-group comparisons. One-way analysis of variance (ANOVA) followed by a post hoc Tukey test was used for multiple groups comparisons. The randomized block ANOVA (repeated measures ANOVA) was used for western blot data with a control value of 1 and no SEM as described previously [22]. A two-tailed p value < 0.05 was considered as a statistically significant difference. Data were analyzed using GraphPad Prism 7.0.

## Results

### Abnormal upregulation of RGS4 produces profibrotic effects in cardiac fibroblasts and MI mice

**Fig. 1:**
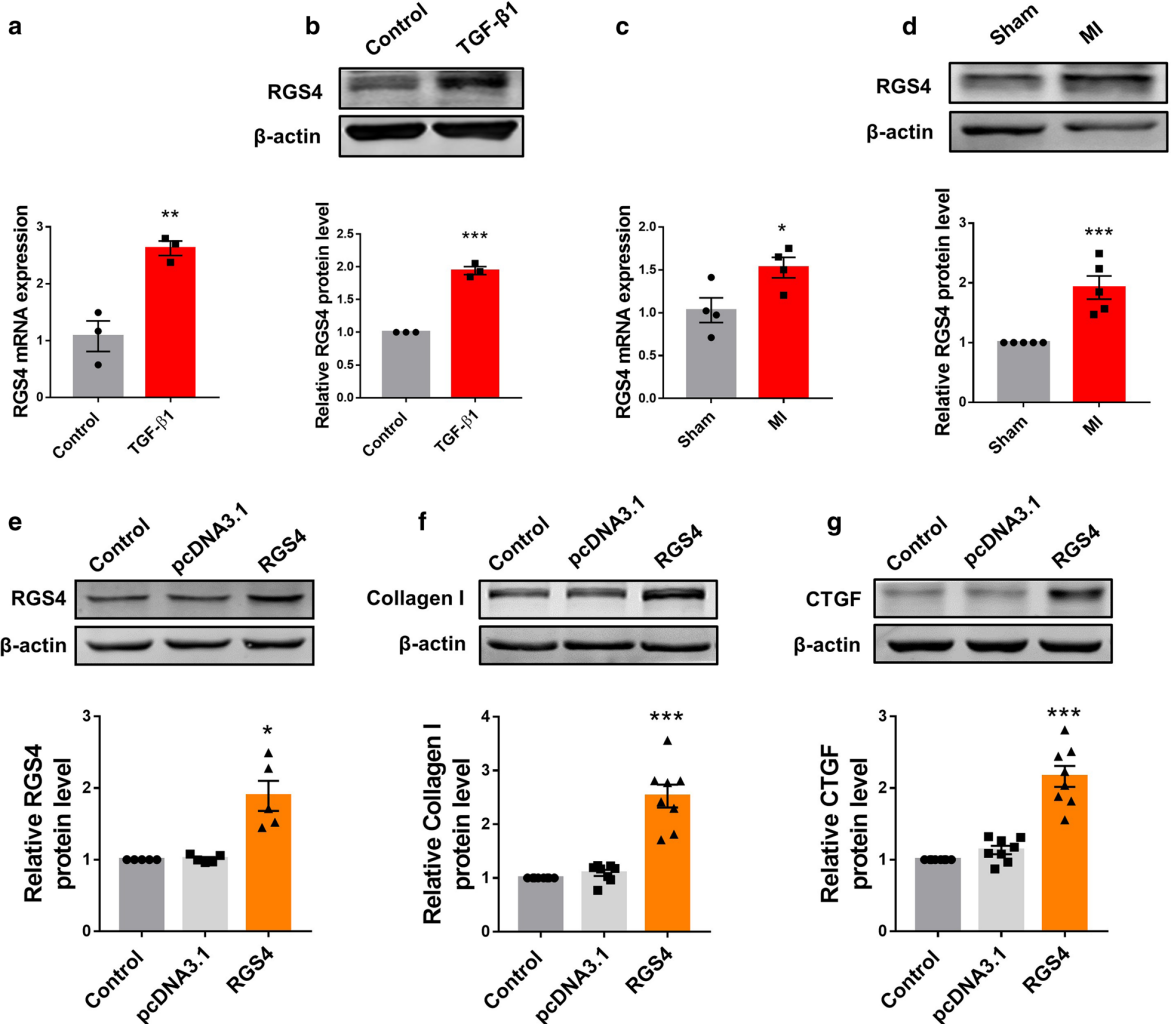
RGS4 is upregulated in TGF-β1-treated cardiac fibroblasts (CFs) and in mice with myocardial infarction (MI). **a** Upregulation of RGS4 expression at both mRNA and **b** protein levels in TGF-β1-stimulated CFs. mRNA level was quantified by qRT-PCR and protein level was determined by Western blot analysis. n = 3. **c** Upregulation of RGS4 expression at both mRNA and **d** protein levels in the heart of mice with MI. n = 4 for mRNA and n = 5 for Western blot. **e** Verification of RGS4 overexpression in CFs transfected with pcDNA3.1 plasmid containing RGS4 gene. **f** Increase in collagen I protein level by RGS4 overexpression. n = 5. **g** Increase in CTGF protein level by RGS4 overexpression. Empty pcDNA3.1 vector serves as a negative control. n = 8 batches of CFs. *p < 0.05, **p < 0.01, ***p < 0.001 versus Control or MI

As the first step towards understanding the potential role of RGS4 in regulating cardiac fibrosis, we assessed the expression of RGS4 in TGF-β1-treated cardiac fibroblasts. As depicted in Fig. 1a, the mRNA level of RGS4 in TGF-β1-treated cardiac fibroblasts was significantly increased compared to the control group. Consistently, the protein level of RGS4 was also higher in the TGF-β1 group than in the control group (Fig. 1b). Similarly, both mRNA and protein levels of RGS4 in MI mice were higher than in the sham-operated control counterparts (Fig. 1c, d).

Next, we examined the effect of RGS4 overexpression on cardiac fibroblasts. Successful overexpression of RGS4 in cardiac fibroblasts with transfection of RGS4 plasmid was first verified (Fig. 1e). Notably, RGS4 overexpression sharply increased the protein levels of collagen I and CTGF, main markers of cardiac fibrosis, were with relative to empty plasmid as a negative control (pcDNA3.1) (Fig. 1f, g).

### Silencing RGS4 mitigates fibrogenesis in cardiac fibroblasts and MI mice

**Fig. 2:**
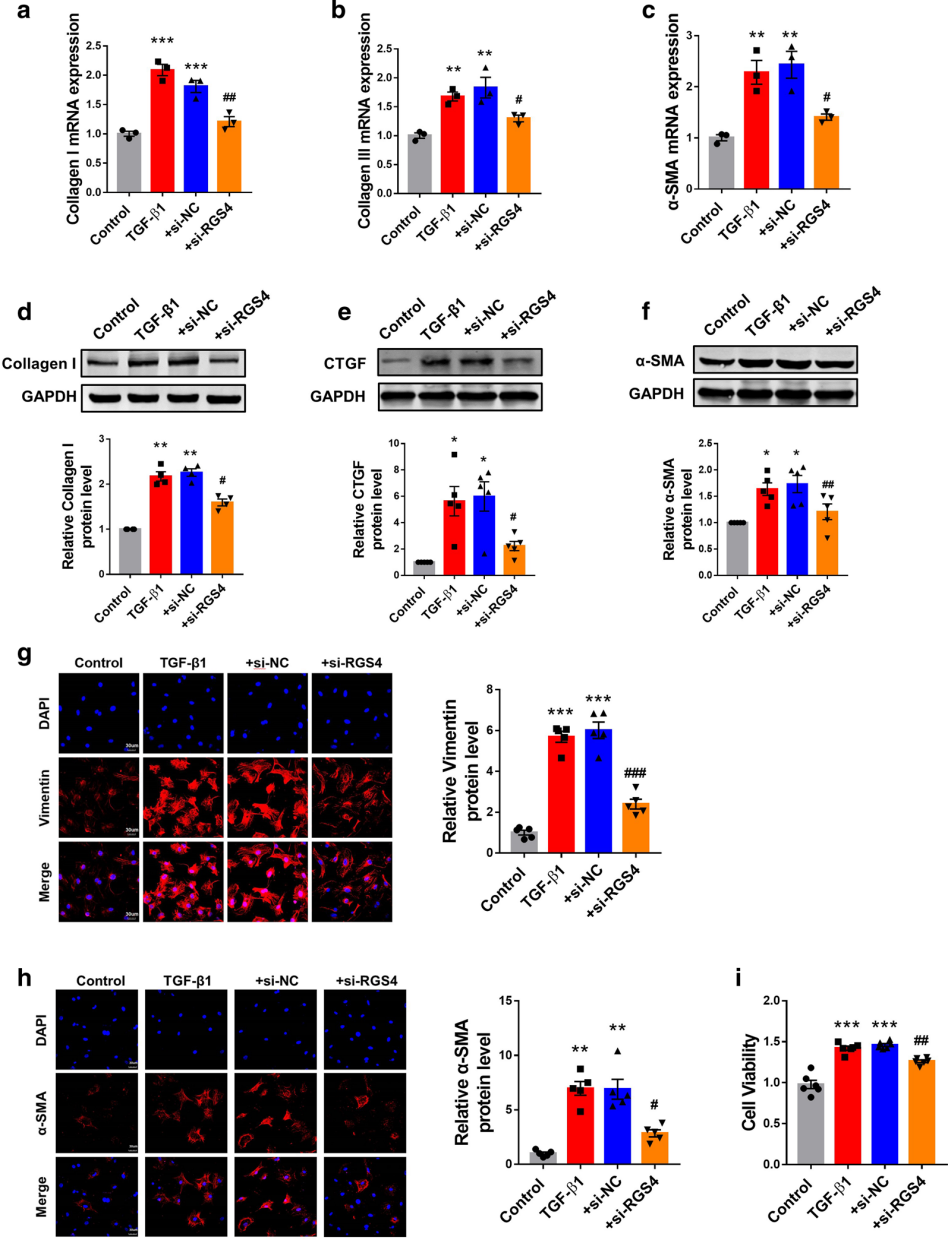
Silencing of RGS4 mitigates cardiac fibrosis in vitro. **a** Silence of RGS4 by siRNA (si-RGS4) suppresses the abnormal upregulation of collagen I, **b** collagen III and **c** α-SMA expression at mRNA level in TGF-β1-stimulated CFs. mRNA level was quantified by qRT-PCR. n = 3. **d** si-RGS4 suppresses the abnormal upregulation of collagen I, **e** CTGF and **f** α-SMA expression at protein level in TGF-β1-stimulated CFs. Protein level was determined by Western blot analysis. n = 4 or n = 5. **e** Left: representative immunostaining images showing the effects of si-RGS4 on the expression of vimentin in TGF-β1-treated CFs as an indication of fibroblast-myofibroblast transition. Right: statistical results of immunostaining expressed as mean ± SEM. n = 5. **f** Left: representative immunostaining images showing the effects of si-RGS4 on the expression of α-SMA in TGF-β1-treated CFs as an indication of fibroblast-myofibroblast transition. Right: statistical results of immunostaining. n = 5. **g** Effect of si-RGS4 on the viability of CFs in the presence of TGF-β1 as measured by MTT assay. n = 6. TGF-β1 + si-NC served as a negative control. *p < 0.05, **p < 0.01, ***p < 0.001 versus Control, ^#^*p* < 0.05, ^##^*p* < 0.01, ^###^*p* < 0.001 versus TGF-β1 + si-NC

While the above results indicated a profibrotic effect of RGS4, we sought to continue our investigation using a loss-of-function approach. To this end, we silenced RGS4 using siRNA (si-RGS4) in cardiac fibroblasts (Additional file 1: Fig. S1A). As anticipated, silence of RGS4 mitigated the TGF-β1-induced abnormal upregulation of collagens I and III and α-SMA at the mRNA level (Fig. 2a–c). Similarly, si-RGS4 also abrogated the increases in the protein levels of collagen I (Fig. 2d), CTGF (Fig. 2e) and α-SMA (Fig. 2f) in the presence of TGF-β1 stimulation.

We then turned to look at whether RGS4 is involved in regulating activation of myofibroblasts. The immunofluorescence staining showed that the TGF-β1 treatment induced an increase of Vimentin and α-SMA which was significantly weakened after RGS4 silencing, indicating a mitigation of fibroblast-myofibroblast transition induced by TGF-β1 (Fig. 2g, h). Moreover, TGF-β1 treatment increased viability of cardiac fibroblasts, as revealed by MTT assay, whereas si-RGS4 suppressed the effect of TGF-β1 (Fig. 2i). It was indicated RGS4 was involved in regulating proliferation of cardiac fibroblasts.

**Fig. 3:**
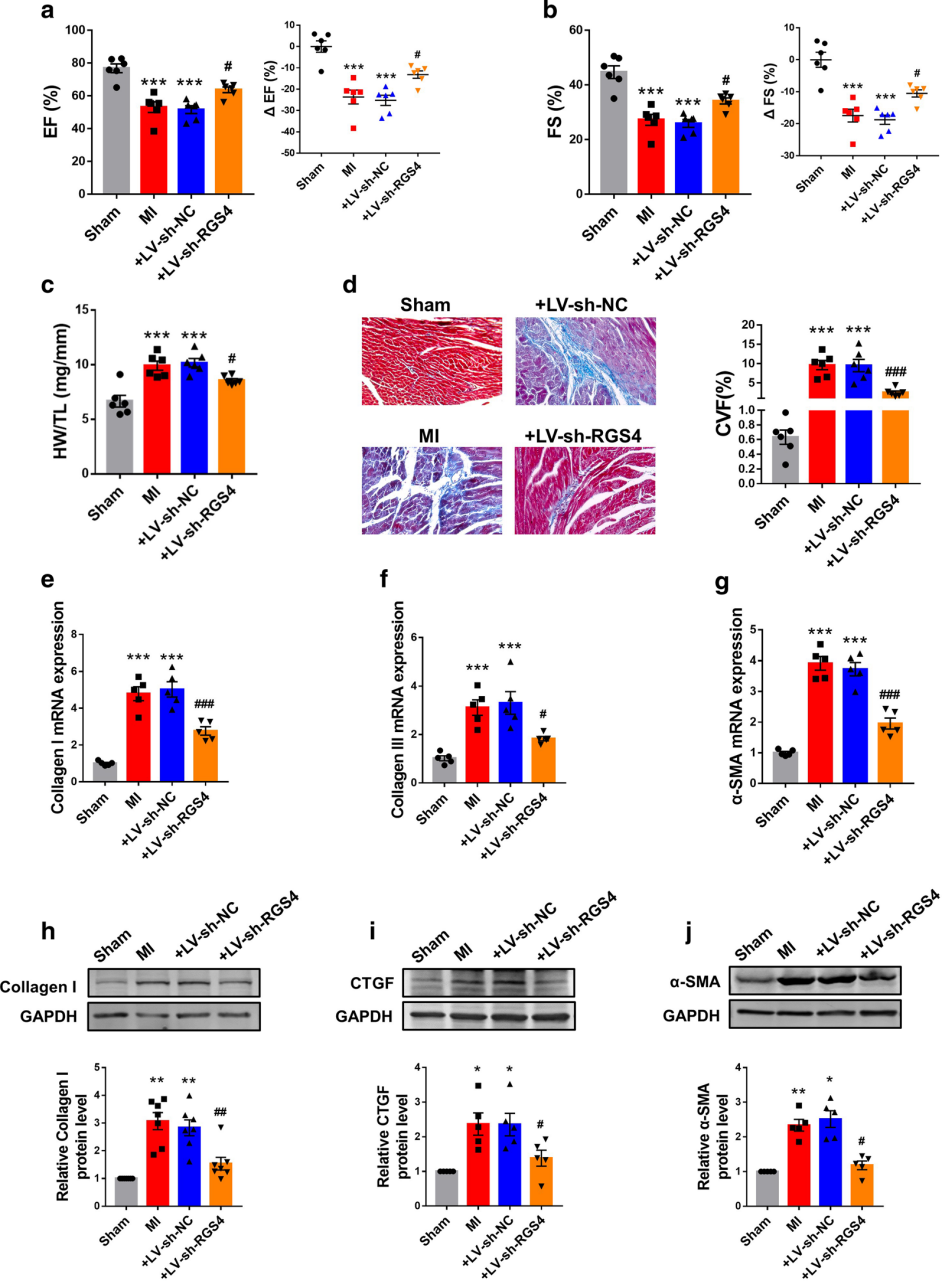
Silence of RGS4 reduces cardiac fibrosis in MI mice. **a** Silence of RGS4 by the lentivirus vector carrying a RGS4-specific shRNA (LV-sh-RGS4) mitigates the MI-induced decrease in ejection fraction (EF%) assessed by echocardiography. Note the lack of effect on LV-sh-NC as a negative control. n = 6. **b** Silence of RGS4 by LV-sh-RGS4 mitigates the MI-induced decrease in fractional shortening (FS%) assessed by echocardiography. n = 6. **c** Silence of RGS4 by LV-sh-RGS4 reduces the MI-induced increase in the ratio of heart weight to tibia length, indicating a relief of hypertrophic response to MI. n = 6. **d** Left: representative Masson-staining images of cardiac sections showing the amelioration of cardiac fibrosis by LV-sh-RGS4 in MI mice relative to the sham-operated control mice. Right: statistical results of Masson staining. CVF: volume fraction of collagen. n = 5. **e** qRT-PCR results demonstrate the diminishment of MI-induced upregulation of collagens I, **f** collagen III and **g** α-SMA mRNA levels in MI mice. n = 3. **h** Western blot results demonstrate the reversal of MI-induced upregulation of collagens I, **i** CTGF and **j** α-SMA protein levels in MI mice I + LV-sh-NC served as a negative control. n = 5 or n = 7. *p < 0.05, **p < 0.01, ***p < 0.001 versus Sham, ^#^*p* < 0.05, ^##^*p* < 0.01, ^###^*p* < 0.001 versus MI + LV-sh-NC

The above results indicate that silence of profibrotic RGS4 could well prevent or reverse cardiac fibrosis. We therefore continued to test the effect of RGS4 knockdown on cardiac fibrosis in MI mice. First, we fist confirmed the successful establishment of the mouse model of RGS4-knockdown through injection lentivirus carrying the RGS4-specific shRNA fragment (Additional file 1: Fig. S1B). Echocardiographic examination showed that ejection fraction (EF%) and fractional shortening (FS%) were both markedly depressed in MI mice relative to sham-operated control littermates (Fig. 3a, b). Silence of RGS4 rescued the impaired cardiac function with significant recovery of EF% and FS% (Fig. 3a, b), and the negative control construct did not produce any appreciable effect. On the other hand, the ratio of heart weight over tibia length was significantly increased in MI heart and RGS4 knockdown abolished this increase (Fig. 3c). Masson’s staining disclosed large amount of collagen deposition in MI mice compared with sham mice, which was improved by RGS4 silence (Fig. 3d). H&E staining showed that the cardiomyocytes in MI group were arranged disorderly and loosely connected, with obviously elevated interstitial inflammatory exudation. However, downregulation of RGS4 expression reduced the injury (Additional file 3: Fig. S3A). Consistently, inhibition of RGS4 expression reduced the increases of expression of cardiac fibrosis markers genes in MI (Fig. 3e–g). In addition, the protein levels of cardiac fibrosis related factors also reflected that inhibition of RGS4 reversed cardiac fibrosis (Fig. 3h–j).

### RGS4 overexpression attenuates the anti-fibrogenic effect of choline in CFs and MI mice

**Fig. 4:**
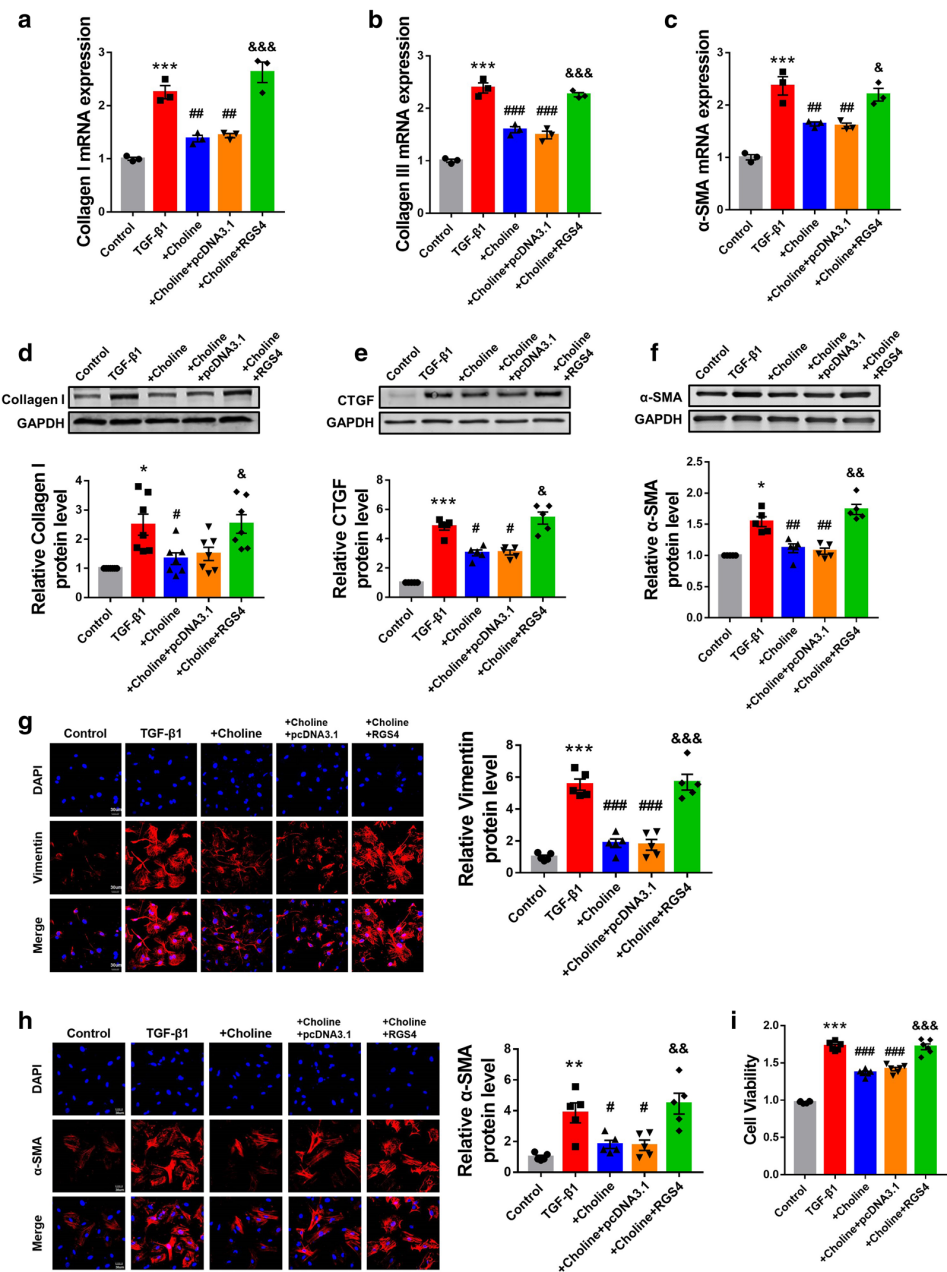
RGS4 attenuates the effect of choline on TGF-β1-induced fibrogenesis in CFs. **a** mRNA expression of Collagen I, **b** Collagen III and **c** α-SMA in CFs was measured by qRT-PCR. n = 3. **d** Protein levels of Collagen I, **e** CTGF and **f** α-SMA in CFs was assessed by western blot analysis. n = 5 or n = 7. **g** Left: representative immunostaining images show the expression of vimentin in TGF-β1-treated CFs as an indication of fibroblast-myofibroblast transition. Right: statistical results of immunostaining expressed as mean ± SEM. n = 5. **h** Left: representative immunostaining images show the expression of α-SMA in TGF-β1-treated CFs as an indication of fibroblast-myofibroblast transition. Right: statistical results of immunostaining. n = 5. **i** The cell viability of CFs measured by MTT assay. n = 6. TGF-β1 + Choline + pcDNA3.1 served as a negative control. n = 5. *p < 0.05, **p < 0.01, ***p < 0.001 versus pcDNA3.1 or Control, ^#^*p* < 0.05, ^##^*p* < 0.01, ^###^*p* < 0.001 versus TGF-β1, ^&^*p* < 0.05, ^&&^*p* < 0.01, ^&&&^*p* < 0.001 versus TGF-β1 + Choline + pcDNA3.1

While the above data strongly suggest the pro-fibrotic action of RGS4, it is speculated that as a negative regulator of G protein signal system, RGS4 may act through blocking G protein-coupled receptors (GPCRs) to stimulate cardiac fibrosis, since it has been documented that choline possesses anti-fibrotic property via activating GRCPs in heart [19]. Therefore, we used choline to verify whether RGS4 works through GRCPs. As illustrated in Fig. 4a–f, choline reduced the TGF-β1-induced increase in mRNA levels of collagens I/III and CTGF (Fig. 4a–c) and protein levels of collagens I, CTGF and α-SMA (Fig. 4d–f) in CFs and strikingly RGS4 overexpression with the pcDNA3.1 plasmid carrying the RGS4 gene surmounted the beneficial effects of choline and regained the abnormal increases of expression of fibrotic marker genes. In addition, as shown by immunofluorescence staining in Fig. 4g, h, RGS4 counteracted the downregulation of vimentin and α-SMA, respectively, by choline. In other words, choline ameliorated fibroblast-myofibroblast transition in CFs induced by TGF-β1, but enhanced expression of RGS4 strengthened fibroblast-myofibroblast transition again. Moreover, choline suppressed CF proliferation as indicated by the alleviation of TGF-β1-stimulated increase in cell viability, and the beneficial effect of choline was abrogated by RGS4 overexpression (Fig. 4i).

**Fig. 5:**
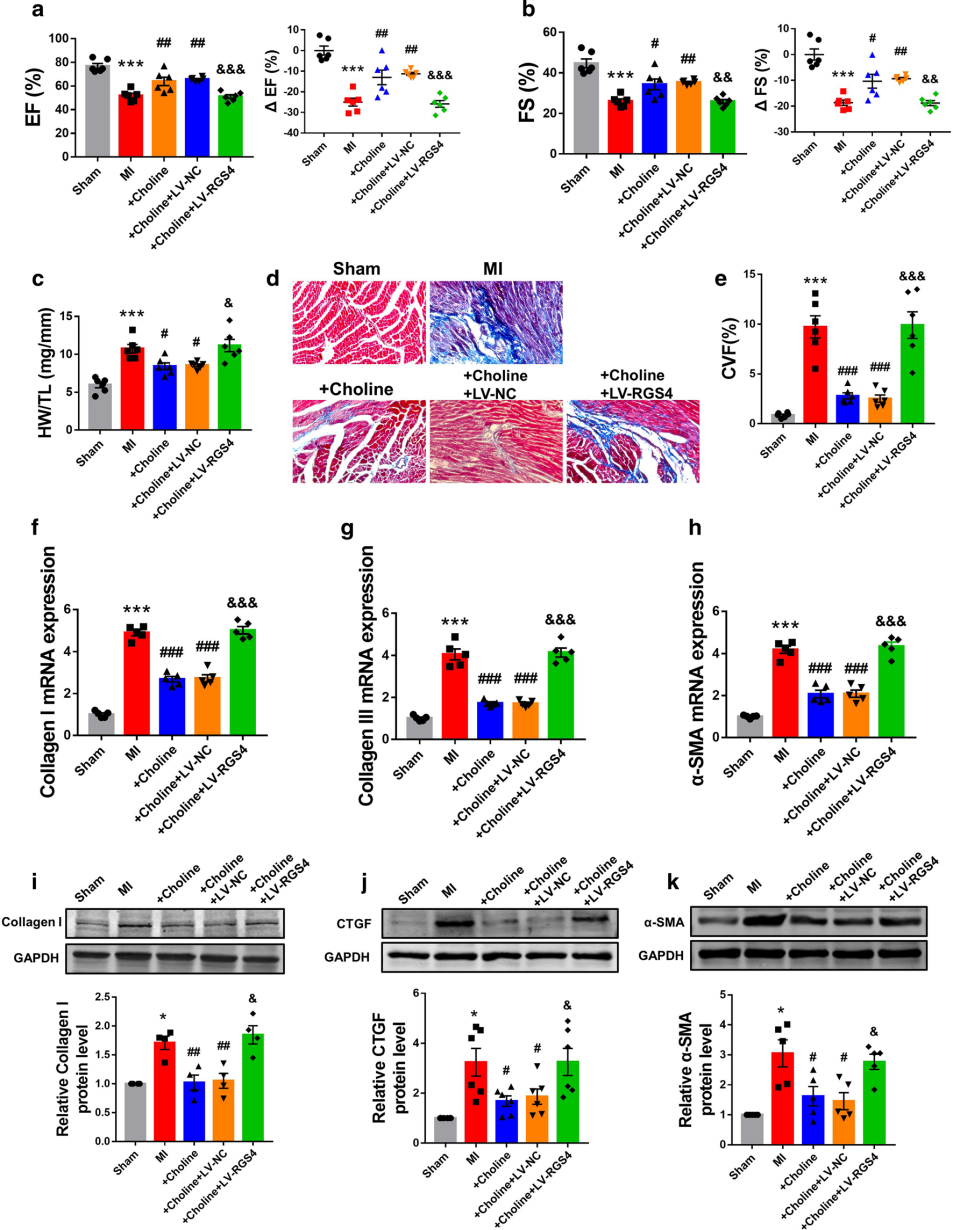
RGS4 as a negative regulator for choline in cardiac fibrosis during MI. **a**, **b** The function of infarcted heart measured by echocardiography. n = 6. **c** The ratio of heart weight and the length of tibia. n = 6. **d** Left: Representative images of Masson-stained hearts of MI mice. Right: Statistical results of Masson staining. CVF: volume fraction of collagen. n = 5. **e** mRNA expression of collagen I, **f** collagen III and **g** α-SMA in MI mice was measured by qRT-PCR. n = 3. **h** Protein levels of collagen I, **i** CTGF and **j** α-SMA in MI mice was assessed by western blot analysis. MI + Choline + LV-NC served as a negative control. n = 5. *p < 0.05, **p < 0.01, ***p < 0.001 versus Sham, ^#^*p* < 0.05, ^##^*p* < 0.01, ^###^*p* < 0.001 versus MI, ^&^*p* < 0.05, ^&&^*p* < 0.01, ^&&&^*p* < 0.001 versus MI + Choline + LV-NC

To see if the in vitro results on RGS4 and choline could be reproduced under in vivo conditions, we went on to evaluate the effects of RGS4 on the cardioprotective action of choline in a mouse model of MI. We successfully overexpressed RGS4 by lentivirus injection firstly (Additional file 1: Fig. S1C). As depicted in Fig. 5a, b, choline normalized cardiac dysfunction induced by MI as indicated by improved EF% and FS%; however, these beneficial effects were antagonized by RGS4 overexpression. The ratio of heart weight to tibia length was reduced by choline in MI mice, and this reduction was abolished by RGS4 overexpression (Fig. 5c). Moreover, Masson staining unraveled that the alleviation of MI-induced collagen deposition by choline was mitigated by RGS4 overexpression (Fig. 5d). H&E staining revealed that choline improved myocardial injury, but overexpression of RGS4 inhibited the effect of choline (Additional file 1: Fig. S3B). Meanwhile, choline inhibited the MI-induced upregulation of cardiac fibrosis marker genes at both mRNA and protein levels, and RGS4 countered these anti-fibrotic effects (Fig. 5e–j).

### RGS4 weakens the anti-fibrotic effect of choline and downstream TGF-β1/Smad and MAPK signaling

**Fig. 6:**
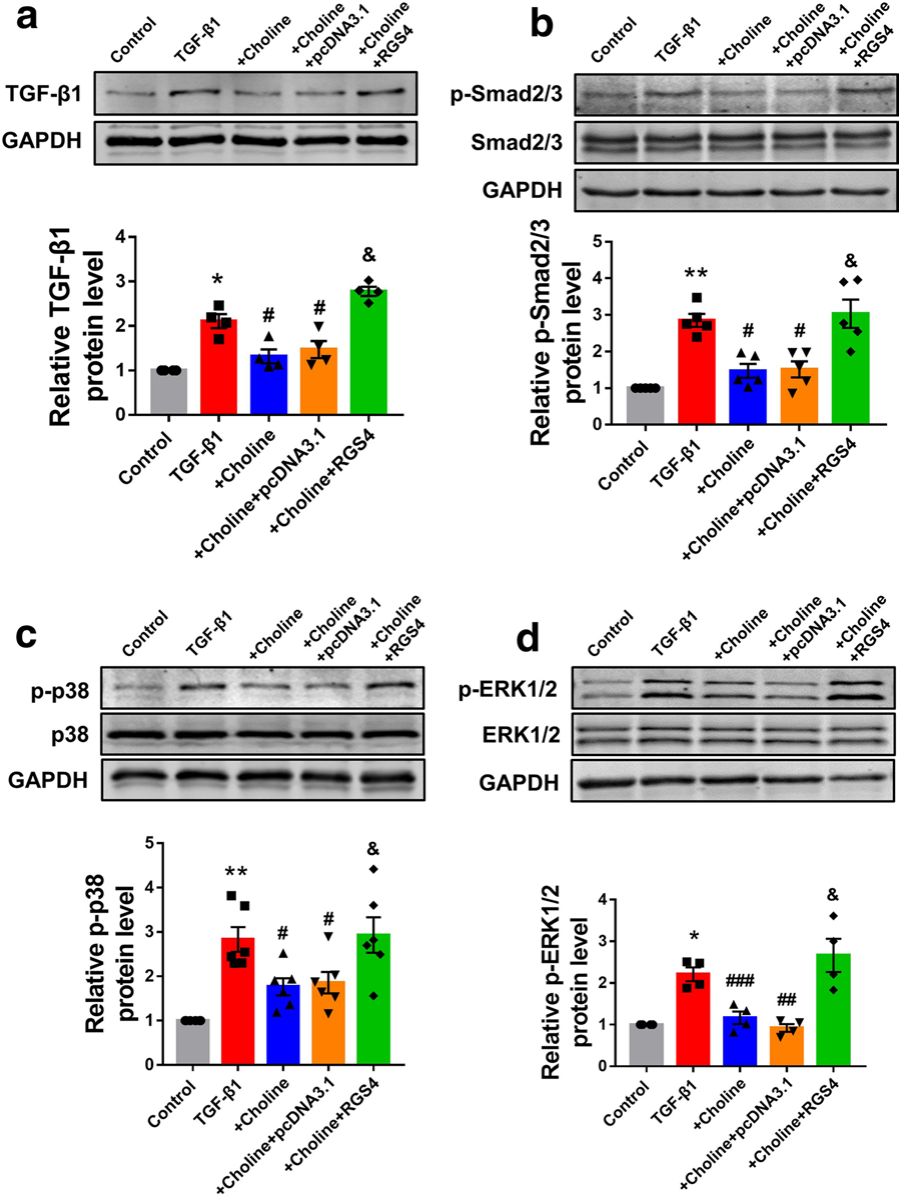
RGS4 antagonized anti-cardiac fibrosis effect of choline through TGF-β1/Smad and MAPK signaling. **a** The protein levels of TGF-β1 and **b** p-Smad2/3, central members of TGF-β1/Smad signaling, was assessed by western blot analysis. n = 4 or n = 5. **c** The protein levels of p-p38 and **d** p-ERK1/2, key members of MAPK signaling, was assessed by western blot analysis. TGF-β1 + Choline + pcDNA3.1served as a negative control. n = 6 or n = 4. *p < 0.05, **p < 0.01 versus Control, ^#^*p* < 0.05, ^##^*p* < 0.01, ^###^*p* < 0.001 versus TGF-β1, ^&^*p* < 0.05 versus TGF-β1 + Choline + pcDNA3.1

It has been demonstrated that choline regulated cardiac fibrosis by suppressing TGF-β1/Smad and MAPK signaling [19]. We therefore next, explored whether RGS4 inhibited the effect of choline through TGF-β1/Smad and MAPK signaling. Our results showed that the protein levels of TGF-β1 and Smad2/3 in the choline group were significantly lower than those in the TGF-β1 group (Fig. 6a, b). The ratio of p-p38/t-p38 and of p-ERK1/2/t-ERK1/2 in the choline group were sharply lower than in the TGF-β1 group, while RGS4 abolished the suppressive effect of choline (Fig. 6c, d).

## Discussion

In this study, we uncovered the pro-fibrotic property of RGS4 in both cellular and whole animal models of cardiac fibrosis with TGF-β1stimulated CFs and MI mice, respectively. Silence of RGS4 reduced the mRNA and protein levels of cardiac fibrosis-related genes, inhibited the abnormal proliferation of CFs and curbed the transformation of fibroblasts into myofibroblasts. Consistently, silence of RGS4 also improved cardiac function and reduced collagen deposition in MI mice. In addition, RGS4 overexpression diminished the anti-cardiac fibrosis effect of choline.

Previous studies have reported that other RGS proteins were involved in cardiac fibrosis. In RGS14 transgenic mice, the extent of aortic banding-induced cardiac fibrosis was exacerbated through MEK-ERK1/2 signaling [12]. Overexpression of RGS2 negatively regulated cardiac fibroblast proliferation and total collagen production induced by angiotensin II, while inhibition of RGS2 by siRNA aggravated cardiac fibrosis [23]. Transgenic mice with cardiac-specific overexpression of human Rgs5 gene were resistant to cardiac hypertrophy and fibrosis via inhibition of MEK-ERK1/2 signaling [24]. The above evidence showed that some RGS proteins played a role in protecting cardiac fibrosis, and our study confirmed that RGS4 promoted cardiac fibrosis. It will be our future work to study the whole effect of RGS proteins and to explore the balance of RGS proteins on cardiac fibrosis.

RGS family members, including RGS4, played a role in accelerating the termination G protein signal transduction by acting as GTPase-activating proteins. The physiological and pathological effect of RGS proteins expression abnormality was through acting on GPCRs such as muscarinic receptors. It was demonstrated that voltage-dependence of RGS4 modulation was derived from the M_2_ muscarinic receptor [25]. Decreased RGS6 caused irregular cardiac rhythmicity and raised susceptibility to atrial fibrillation, which was contributed by m_2_R-I_KACh_ intracellular signaling pathway [26]. Both in neonatal atrial myocytes and adult sinoatrial nodal cells, RGS6 downregulation contributed profound delays in m_2_R-I_KACh_ deactivation kinetics [27]. RGS4 inhibits the release of insulin mediated through beta-cell M_3_ muscarinic receptors by binding to M_3_ receptor to form complex in type 2 diabetes [11]. In addition, spinophilin (SPL), a multidomain scaffolding protein, posed as RGS4 agonist to recruit RGS4 into the M_3_R signaling complex to inhibit insulin release [28]. It could be seen from the Figs. 4 and 5 in our research that RGS4 weakened the protective effect of choline on cardiac fibrosis both in vivo and vitro, in other words, RGS4 affected the process of cardiac fibrosis by mediating cholinergic receptors.

It can be seen that RGS4 inhibited the function of M3R and affected the TGF-β/Smad and MAPK pathways in cardiac fibrosis. According to previous studies, we found that the occurrence and development of cardiac fibrosis are closely related to oxidative stress, so we explored the relationship between RGS4 and oxidative stress in cardiac fibrosis [29–32]. As depicted in Additional file 2: Fig. S2A, MI decreased the levels of superoxide dismutase (SOD) activity in heart tissue as compared to Sham group. As expected, the downregulation of SOD activity induced by MI injury was blocked by silencing RGS4 expression. The content of malondialdehyde (MDA) in heart tissue was increased obviously in MI mice, which was significantly attenuated after the expression of RGS4 decreased (Additional file 2: Fig. S2B). As shown in the Additional file 2: Fig. S2C and D, choline attenuated oxidative stress in heart tissue of MI mice, but choline’s effect disappeared after overexpression of RGS4. Oxidative stress produces a lot of reactive oxygen species (ROS) which are reactive chemical species containing oxygen. ROS derived from NADPH oxidase and located within cell membranes, mitochondria, peroxisomes, and endoplasmic reticulum [33, 34]. Cardiac fibroblasts use mitochondrial ROS as a second messenger to promote different signal transduction pathways in cardiac fibrosis, such as TGF-β/Smad pathway [35]. ROS was required to participate in TGF-β induced differentiation of cardiac fibroblasts [32, 36]. Nox4 NADPH oxidase may be an important downstream effector of TGF-β induced cardiac fibrosis, in addition to NADPH oxidase dependent redox signal may in turn regulate TGF-β/Smad signal in a feedforward manner [34, 37, 38]. MAPK pathway may be more closely related to ROS. ROS directly acted on ERK1/2, p38 and JNK, which leads to the formation of a large number of extracellular matrix proteins and aggravates cardiac fibrosis [39]. Based on the above evidence, oxidative stress involved RGS4 in the regulation of cardiac fibrosis.

Choline, as a dietary nutrient, also plays an important role in cardiovascular disease. Choline, as a vitamin B complex factor, is an essential nutrient, although strictly not a true vitamin. Choline plays an important physiological role in the development and function of the cardiovascular system. Choline deficiency is associated with significant cardiovascular incidence rate and even death rate [40, 41]. According to Athina a. strilakou et al., choline deficiency has an adverse effect on cardiac function. After choline deficiency diet in Wistar rats, left ventricular developed pressure (LVDP) results showed that the left ventricular diastolic function was impaired, the serum brain natriuretic peptide (BNP) concentration was increased, and lymphocyte infiltration in myocardial and valve was increased. Obviously, choline deficiency could lead to the damage of cardiac function [41]. Choline also plays an important role in heart development. A higher proportion of heart defects were found in embryos from female rats fed a choline deficient diet, and these defects were often ventricular septal defects (VSD). Adequate intake of choline may promote mouse embryonic growth and heart development, and may also prevent pregnancy syndrome [42]. Carnitine, as a chemical analogue of choline, also has certain cardioprotection like choline. Cardiac damage caused by choline deficiency diet in adult rats could be antagonized by carnitine. Carnitine significantly improved myocardium contractility, diastolic left ventricular function and serum BNP concentration, which are caused by choline deficiency diet [41].

There were multiple GPCRs in the heart, including adenosine, adrenomedullin, angiotensin II, apelin, bradykinin, corticotropin-releasing hormone, endothelin 1, glucagon, histamine, muscarinic acetylcholine, prostanoid, relaxin, serotonin and vasopressin receptors, whose functions were affected by RGS proteins [43, 44]. In this study, RGS4 also affected many GPCRs at the same time when cardiac fibrosis occurred and RGS4 affected GPCRs which could be activated by choline. It will be our future aims at how RGS4 affects other GPCRs and the overall effect of RGS4 interaction with these GPCRs on cardiac fibrosis.

## Conclusion

RGS4 promotes cardiac fibrosis and attenuates the anti-cardiac fibrosis of choline. RGS4 may weaken anti-cardiac fibrosis of choline through TGF-β1/Smad and MAPK signaling pathways.

## Supplementary information

**Additional file 1.** The RGS4 protein level after knocking down and overexpression.

**Additional file 2.** The effect of RGS4 on oxidative stress in MI mice.

**Additional file 3.** Hematoxylin-eosin (H&E)-staining of heart tissue in MI mice.

**Additional file 4.** Full protein bands.

## Data Availability

The datasets used and/or analysed during the current study are available from the corresponding author on reasonable request.

## Abbreviations

IHD: Ischemic heart disease
MI: Myocardial infarction
TGF-β1: Transforming growth factor-β1
RGS: Regulators of G protein signaling
GPCRs: G protein-coupled receptors
CFs: Cardiac fibroblasts
EF%: Ejection fraction
FS%: Fractional shortening
GAPs: GTPase-activating proteins
CVF: Volume fraction of collagen
