# Supplementary material for: The association between RGS4 and choline in cardiac fibrosis
Source: Cell Commun Signal. 2021 Apr 23;19:46. doi: 10.1186/s12964-020-00682-y (PMC8063380; doi:10.1186/s12964-020-00682-y)
Supplement: Supplementary file 2 — Additional file 1. The RGS4 protein level after knocking down and overexpression. [file 12964_2020_682_MOESM2_ESM.pdf]

**S1: The RGS4 protein level after knocking down and over expression.**

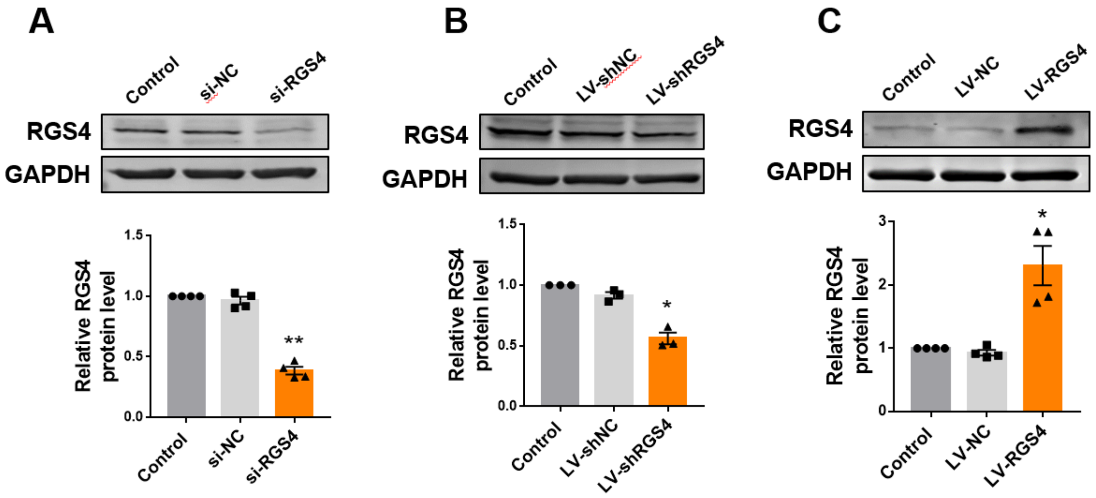


S1. (A) Protein level of RGS4 in CFs was assessed by western blot analysis. n=4. ***p*<0.01 *vs.* si-NC. (B) Protein level of RGS4 in left ventricle 7 days after lentivirus injection shRNA was detected by western blot analysis. n=3. **p*<0.05 *vs.* LV-shNC. (C) Protein level of RGS4 in left ventricle 7 days after lentivirus injection was detected by western blot analysis. n=4. **p*<0.05 *vs.* LV-NC.
