# Supplementary material for: The association between RGS4 and choline in cardiac fibrosis
Source: Cell Commun Signal. 2021 Apr 23;19:46. doi: 10.1186/s12964-020-00682-y (PMC8063380; doi:10.1186/s12964-020-00682-y)
Supplement: Supplementary file 3 — Additional file 2. The effect of RGS4 on oxidative stress in MI mice. [file 12964_2020_682_MOESM3_ESM.pdf]

S2: The effect of RGS4 on oxidative stress in MI mice.

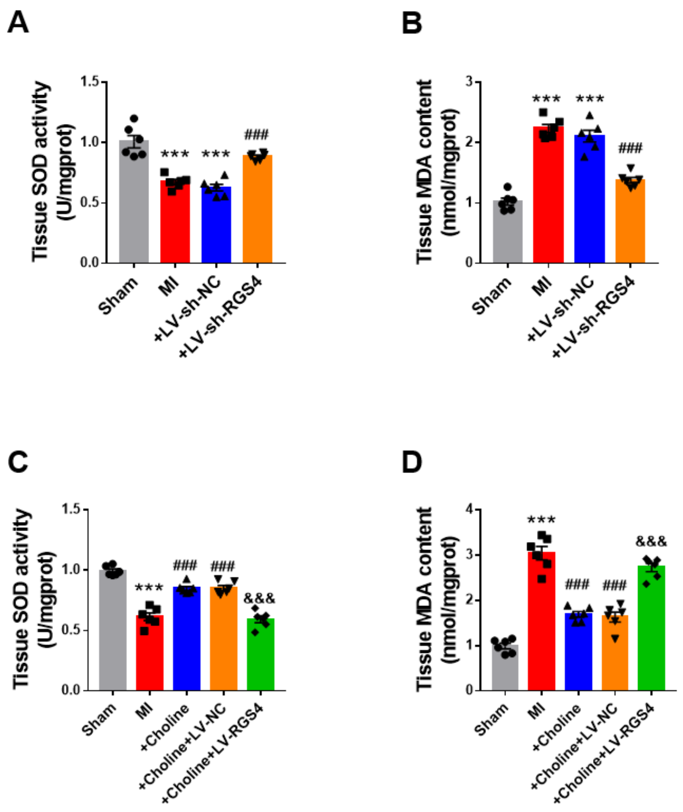

S2. (A) Cardiac tissue SOD activity. n = 6. (B) Cardiac tissue MDA content. n = 6. $^{***}p<0.001$ *vs*. Sham, $^{\#\#\#}p<0.001$ *vs*. MI+LV-sh-NC. (C) Cardiac tissue SOD activity. n = 6. (D) Cardiac tissue MDA content. n = 6. $^{***}p<0.001$ *vs*. Sham, $^{\#\#\#}p<0.001$ *vs*. MI, $^{\&\&\&}p<0.001$ *vs*. MI+Choline+LV-NC.
