# Supplementary material for: The association between RGS4 and choline in cardiac fibrosis
Source: Cell Commun Signal. 2021 Apr 23;19:46. doi: 10.1186/s12964-020-00682-y (PMC8063380; doi:10.1186/s12964-020-00682-y)
Supplement: Supplementary file 4 — Additional file 3. Hematoxylin-eosin (H&E)-staining of heart tissue in MI mice. [file 12964_2020_682_MOESM4_ESM.pdf]

S3: Hematoxylin eosin (H&E)-staining of heart tissue in MI mice.

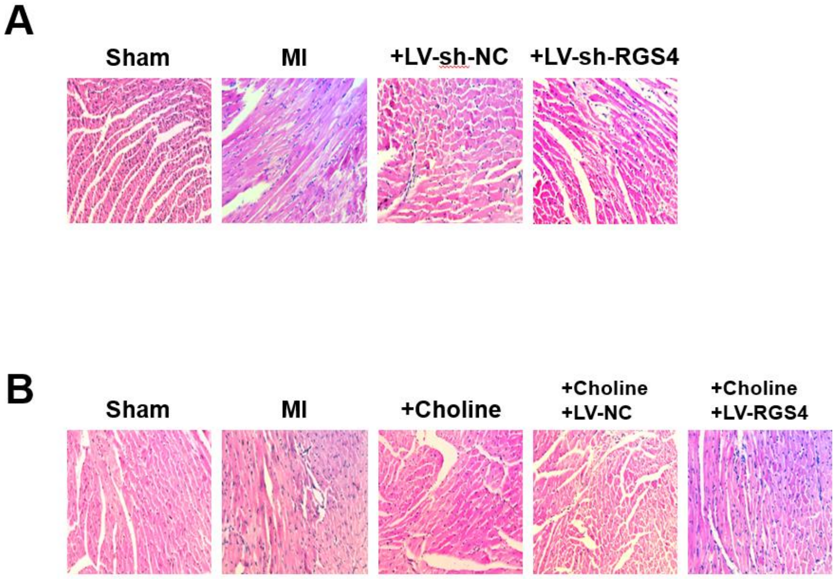

S3. (A), (B) Representative photomicrographs of hematoxylin eosin (H&E)-stained cross-sections of the heart.
