# Supplementary material for: The association between RGS4 and choline in cardiac fibrosis
Source: Cell Commun Signal. 2021 Apr 23;19:46. doi: 10.1186/s12964-020-00682-y (PMC8063380; doi:10.1186/s12964-020-00682-y)

S4: Full protein band

Figure1. B

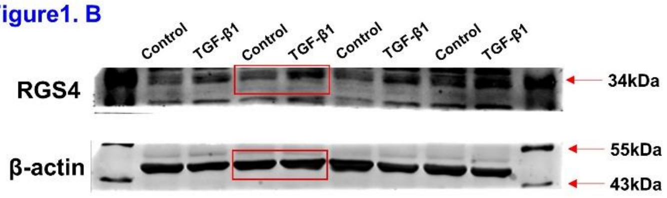

Figure1. D

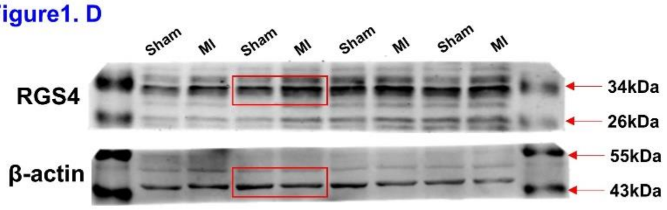

Figure1. E

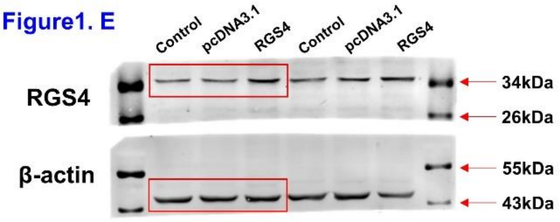

Figure1. F

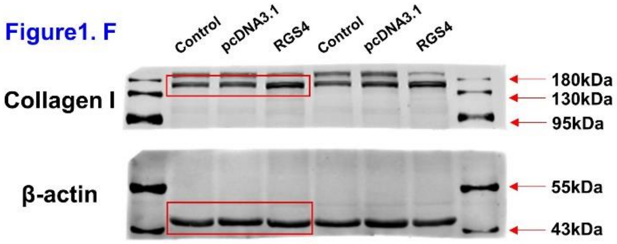

Figure1. G

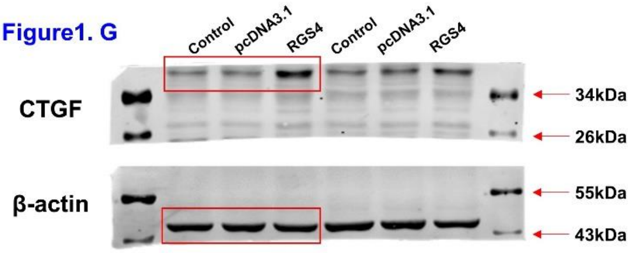

Figure2. D

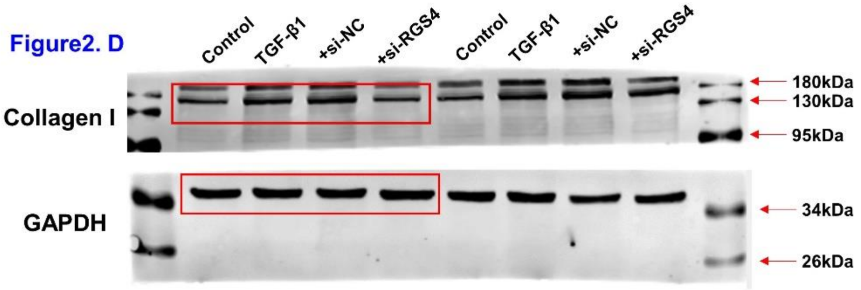

Figure2. E

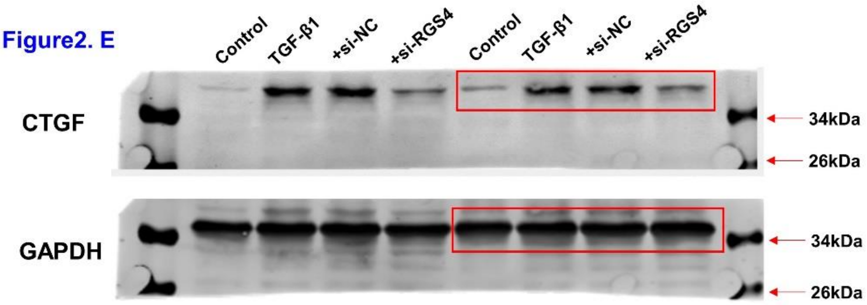

Figure2. F

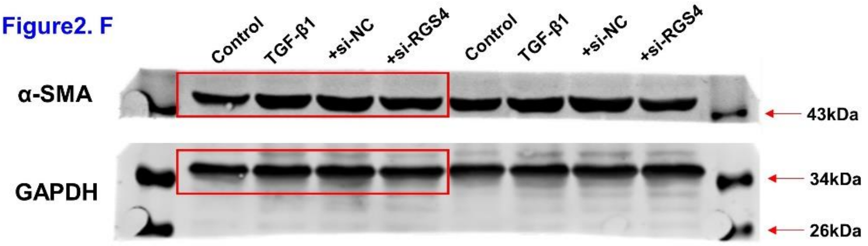

Figure3. H

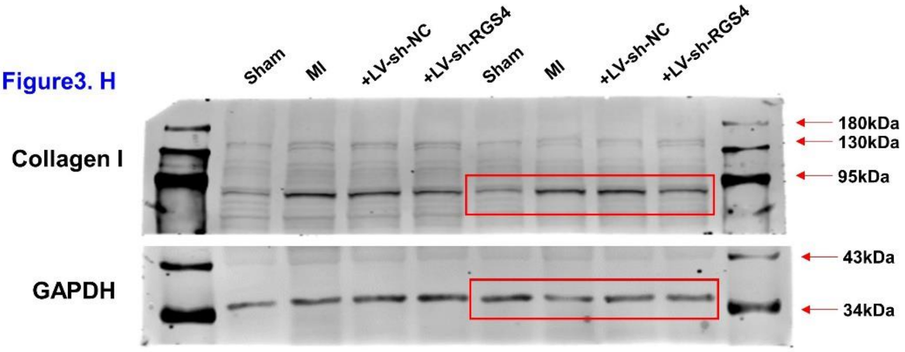

Figure3. I

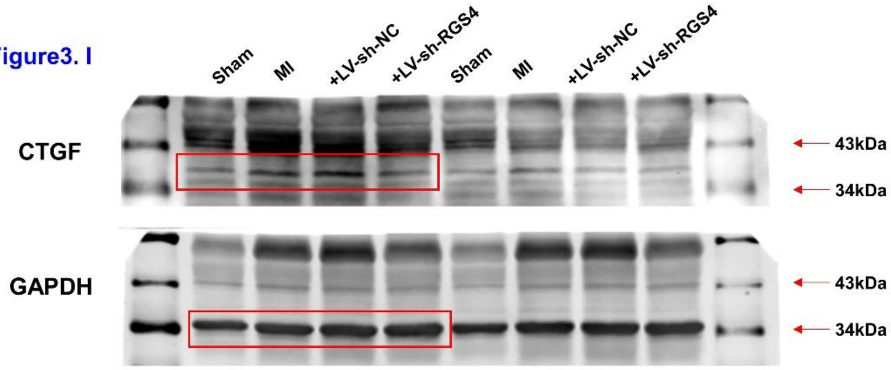

Figure3. J

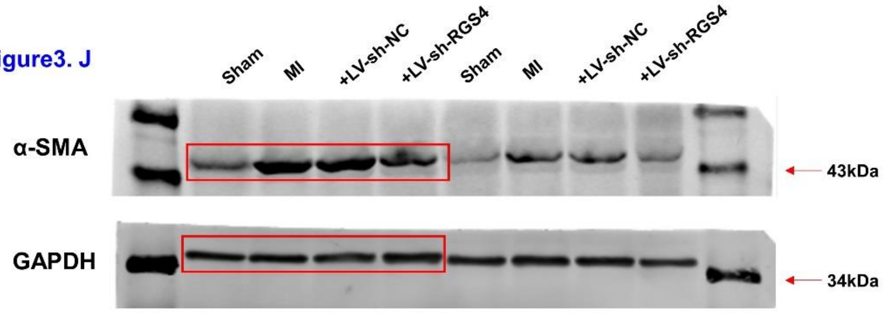

Figure4. D

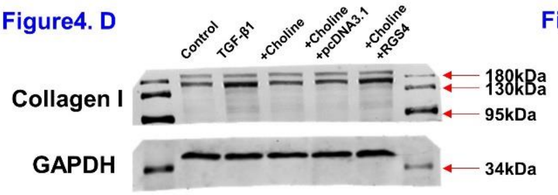

Figure4. E

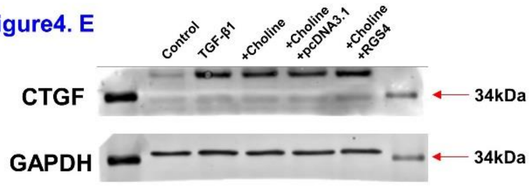

Figure4. F

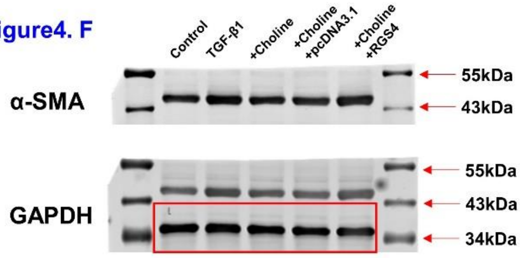

Figure5. I

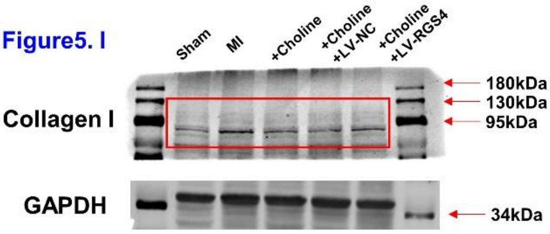

Figure5. J

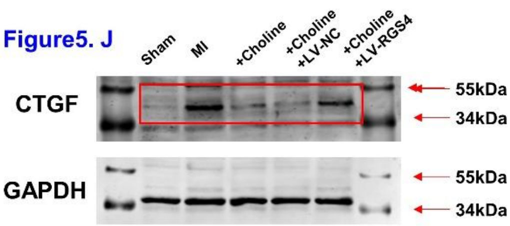

Figure5. K

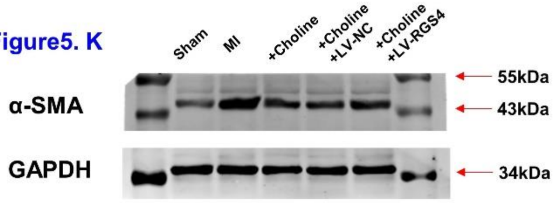

Figure6. A

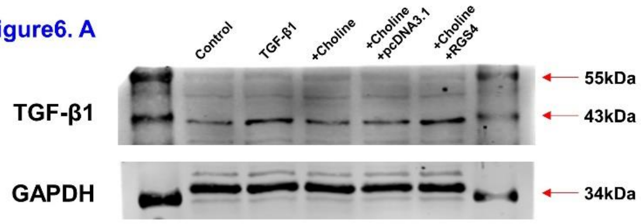

Figure6. B

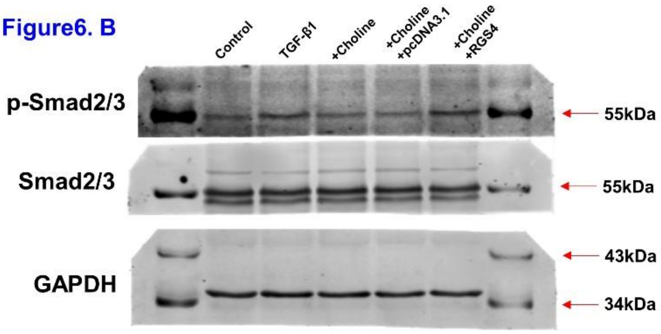

Figure6. C

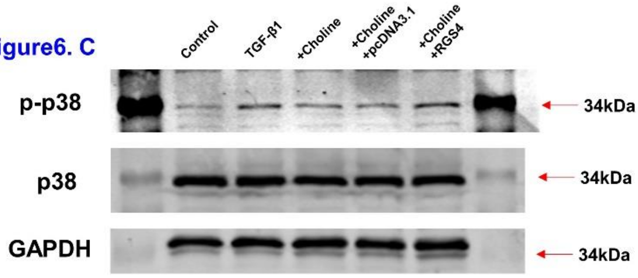

Figure6. D

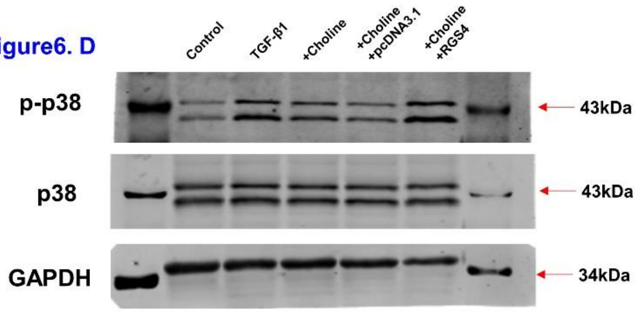

Supplement: Supplementary file 5 — Additional file 4. Full protein bands. [file 12964_2020_682_MOESM5_ESM.pdf]
